# Clinical Report on Chronic Pleurisy, Based on an Analysis of Forty-seven Cases

**Published:** 1852-11

**Authors:** Austin Flint


### BUFFALO MEDICAL JOURNAL

AND

### MONTHLY REVIEW.

**VOL. 8.** NOVEMBER, 1852. **NO. 6.**

### ORIGINAL COMMUNICATIONS.

ART. I.—*Clinical Report on Chronic Pleurisy, based on an analysis of Forty-seven cases.* By AUSTIN FLINT, M. D.

The study of Chronic Pleurisy by means of the analytical investigation of recorded observations, offers a field of scientific research which, as yet, has been cultivated but to a very limited extent. In proof of the correctness of this remark, it is sufficient to refer to the cursory and loose manner in which the subject is treated by the compilers of the most approved systematic works on practical medicine, and even in treatises devoted specially to diseases of the chest. Without professing to have taken pains to institute an extensive bibliographical examination with respect to this point, I am unable to cite any work which presents numerical results deduced from a larger collection of data, than that by Dr. Blakiston.* This author gives the facts elicited by an analysis of seventy-eight cases. His enumerations are confined to a few only of the points pertaining to the natural history of the disease, a very small portion of the work being devoted to the subject. The late Dr.

* Practical Observations on certain Diseases of the Chest, and on the Principles of Auscultation. By Peyton Blakiston, M. D., F. R. S. Republished by Lea & Blanchard, Philadelphia, in 1848.

James Hope, during his last illness, was engaged in arranging the materials for a memoir on this subject, which should embody the fruits of his large experience, and the posthumous publication of the brief notes relating thereto, dictated on the bed of death, tends to enhance regret that he did not live to execute his design, and add another treatise to the works which will render his name ever illustrious in the annals of medical science.

It is needless to say with respect to this, as well as other diseases, that accumulated clinical observations constitute the true basis of its symptomatology, diagnosis, and our knowledge of the laws regulating its development, progress, and results. This is not less clear than the fact that our present knowledge of it in these relations is imperfect. There are several pathological questions of practical interest which remain to be settled by numerical investigations, such as the connection of the chronic with the acute form; its dependence on tuberculosis or other antecedent affections; the consecutive production of Phthisis or other diseases; the ratio of its fatality, and the influence of remedial agencies, separately and combined. On all these points the notions to be derived from current medical literature are wanting in precision, sometimes discrepant, and, as a matter of course, to a greater or less extent, erroneous.

There is another aspect in which the importance of the study of Chronic Pleurisy is strikingly apparent. The disease, although infrequent in its occurrence, is not so rare but that it occasionally falls under the notice of most practitioners of medicine, however restricted the circuit of their observations. But in a very large proportion of instances it is not recognized. As will be seen in the sequel, a great majority of the cases that I have collected, had been under the treatment of one or more physicians who had failed to discover the seat and character of the affection. It is not probable that these cases are notably peculiar in the fact of their escaping diagnosis, for it is remarked by Dr. Hope that "there is no class of affections more habitually overlooked by the bulk of the profession than this." It necessarily follows from this fact, that the disease very frequently fails to receive appropriate management, and, perhaps, nearly as often is treated by measures which are more or less injurious.

The considerations just adduced have led me to examine the facts relating to Chronic Pleurisy contained in the histories of the cases coming under my observation during several years that I have been accustomed to keep notes of public and private practice. The number of cases is not very large, and, on the other hand, not so small as to be insignificant. A more serious defect is the incompleteness of the histories. A considerable proportion of the patients were either seen in consultation, or applied simply for a physical exploration of the chest. Under these circumstances a general sketch of the previous history only was noted, and in several instances the farther progress and issue were not procured. The cases, moreover, came under my notice at different stages of the history, and the notes of some were made after recovery from the disease. The data which I have collected are, thus, manifestly inadequate for the positive and negative results of a complete numerical analysis. To this character the present Report can have no claim. My only expectation is to furnish a small contribution toward the cultivation of a field of clinical study, of great practical interest, which, from the difficulty of aggregating facts, or other causes, has hitherto been neglected.

The number of cases of which I have preserved notes, is *forty-seven.* These cases have occurred, some in hospital and others in private practice, for the most part during the last eighteen years. A few are of an older date. For convenience of consideration and reference, they admit of being distributed into several groups. One of these groups will embrace the cases which came under observation while the disease was progressing either favorably or unfavorably. Another group will include cases in which the diagnosis and history were retrospective, that is to say the facts were obtained at a period more or less distant from the occurrence of the disease, being restricted in a great measure to its consequences or sequelæ. Another will embrace cases of circumscribed, as distinguished from general pleurisy. Another will embrace cases of pleurisy from perforation, constituting the affection known by the incorrect title of pneumo-hydro-thorax. I shall speak of these several groups, excepting the first, under distinct heads, but with reference to some points of inquiry it will be proper to examine the cases collectively, disregarding the foregoing divisions. Before considering these several groups, therefore, distinctly, I shall take up the points just alluded to, devoting separate consideration to the symptomatology, the physical signs and diagnosis, and I shall, in conclusion, offer a few remarks on the management of the disease.

*Age.* Excluding the cases in which the disease occurred subsequently to perforation of the lung, and a single case in which the pleurisy was circumscribed and probably due to external injury, the maximum of age is *forty-five.* One patient, only, was as old as this. One patient was forty-one; one was thirty-eight, and two patients were thirty-five. In all the remaining cases the age was below thirty. The youngest patient was five years of age. Two were of this age. The next youngest was six years; and the next twelve. The average age in thirty-six cases in which the age was ascertained as near as practicable, is twenty-two and a quarter years. The disease, according to these results, is not without the influence of age. It is a disease incident to early life, occurring, in a very large proportion of cases, before thirty, and very rarely after forty years of age.

*Sex.* *Thirty-four* of the cases were males, and *eleven* were females. In the two remaining cases the sex is not noted, the patients being children.

*Season.* Of eighteen cases in which the date of the commencement of the disease is noted, excluding the cases in which the disease was due to perforation, *two* occurred in June; *three* in February; *one* in April; *one* in May; *five* in June; *one in* July; *three* in October, and *two* in December.

*Previous health.* Of twenty-five cases in which the health of the patients prior to the occurrence of the disease was ascertained, it was good in *fifteen.* The cases due to perforation are excluded from this enumeration. In the remaining ten cases in which the previous health was poor, the following are the facts in the cases respectively: One patient had rubeola three months before, with a continuance of cough and debility up to the time of coming under treatment for pleurisy. Another had a severe attack of scarlatina anginosa, a month before coming under observation with pleurisy. In two cases, females, the health had been delicate for some time, with no special malady except leucorrhea; one of these patients had had lichen a year before. Another, a lad, aged fifteen, had been subject, from infancy, to two or three attacks yearly of difficult breathing, considered to be asthma. Another had suffered for several months from a chronic indolent ulcer on the leg. Another had parotitis shortly previous. In *three* cases the history rendered it probable that tuberculosis existed prior to the pleurisy; and in *one* of these cases the pleurisy was preceded by articular inflammation of the ankle joint, of brief duration.

The foregoing results, coupled with the fact that in 3-5 of the cases the previous health of the patients was supposed to be good, go to show that the disease becomes developed without reference to any particular class of affections as antecedents. The small number of cases in which the evidences of tuberculosis were contained in the histories is worthy of notice. It is, indeed, not improbable that tuberculosis may have existed, in some instances, prior to the pleurisy, and the history furnish no distinct evidence of the fact. In no case was the patient subjected to physical exploration to determine the non-existence of phthisis before coming under observation for pleurisy. And, on the other hand, there is the absence of the demonstrative proof of tubercle afforded by the physical signs in the few cases in which it is supposed to have existed. The pre-existence of phthisis is only rendered probable by the rational symptoms entering into the early histories. It seems fairly deducible that, judged by these observations, a very large proportion of cases of Chronic Pleurisy do not involve, as an antecedent affection, tuberculosis. The occurrence of tubercle subsequent to the pleurisy, is an interesting point of inquiry, but this will be deferred for the present.

*Character of the disease at the commencement.* In *six* cases the facts noted with respect to the previous history are insufficient to determine whether the disease was acute, or subacute at the commencement. Excluding these, together with the cases in which the disease was consecutive to perforation, and those in which it was circumscribed, the remaining number of cases is *thirty-five.* Of these thirty-five cases the pleurisy was subacute from the first in *twenty-nine;* it was acute before it became chronic in *two;* and it was subacute at first, after a few days assuming an acute form, and then becoming chronic in *four.* According to these results subacuteness in the inflammatory action from the date of the attack is decidedly the rule with respect to Chronic Pleurisy. This does not agree with the statements generally made in works on practical medicine, and on diseases of the chest, relative to this point. The chronic form of the disease is said usually to follow the acute. To avoid misconception, inasmuch as the terms acute and subacute allow some degree of latitude in their application, it should be added that in all the cases which are considered to have been subacute from the commencement, severe pain occasioning embarrassment in respiration, and high febrile movement were absent. The pain, in fact, was usually slight in degree, and sometimes scarcely present at all, the patients frequently not taking to the bed, or applying for medical aid, till the affection might be considered to have become chronic.

*Seat.* Excluding four cases in which the disease followed perforation, and one case in which it was circumscribed and probably due to mechanical injury, the remaining number is *forty-two.* Of these forty-two cases the disease was seated in the *right* side in *nineteen,* and in the left side in *twenty-three.* The disparity as respects the side affected is not nearly so great in this collection of cases, as in those analyzed by Dr. Blakiston. The latter gave, of seventy-eight cases, the right side affected in *twenty*, and the left in *fifty-eight.*

*Complications.* In three cases the histories render it probable that tuberculosis coexisted. The disease occurred in connection with typhoid fever in one case, and with what was regarded as infantile continued fever in another case. Chronic laryngitis coexisted in two cases, in one of the cases tubercle probably also existing, but not in the other case, as proved by the autopsy.

A chronic ulcer of the leg in one case. Ascites, pericarditis, and a slight degree of chronic inflammation in the other side of the chest, in one case. Pericarditis was supposed from the physical signs to be present, in one case, and was demonstrated after death in another case. These are all the complications that can be ascertained from the histories. The cases in which perforation preceded the pleurisy are excluded.

*Causes.* In four cases the disease was occasioned by perforation of the lung. In two of these cases the perforation occurred in consequence of circumscribed gangrene, and in two instances in connection with tuberculosis. In one case in which the pleurisy was circumscribed, it was probably caused by external injury. This case will be given at length under another head. Of the remaining cases, in the great majority, not even probable causes are mentioned in the histories. It is stated in the history of two cases that the patient was attacked after unusual exertion and exposure. In another instance the patient was attacked suddenly, after exposure to cold. Another patient was seized after getting the feet wet and apparently taking cold. These are all the causes assigned, but it is proper to state that I am by no means certain that pains were generally taken to obtain information from the patients or friends relative to this point. The probable origin of the disease in one of the cases claims particular notice. The patient, a girl, twelve years of age, was attacked suddenly, without any assignable cause, with pain in the back, extending laterally over the right side, but not to the anterior surface of the chest. No medical attendance was obtained until a week afterward, when she was seen by Dr. Josiah Trowbridge, of this city. At that time Dr. T. discovered a swelling situated on the latero-posterior portion of the chest. He regarded it as phlegmonous inflammation, deeply seated, but exterior to the walls of the chest. At the expiration of another week, Dr. Trowbridge saw the patient again, and he then thought there was fluctuation, but did not deem it advisable to make an opening. The swelling was very tender to the touch. A few days after the second visit by Dr. Trowbridge, Dr. Sprague, of this city, was called in. He regarded it in the same light as did Dr. T., and directed poultices, which had already been applied, to be continued. I saw the patient three weeks after the date of the attack. There were, then, the evidences of Chronic Pleurisy, with considerable enlargement of the chest on the right side. In my note of the first examination, nothing is stated relative to the presence of a tumor, and as the case came under observation many years ago, I am unable to say, from recollection, whether it existed or not. If it were present, or in any degree prominent, it is singular that in the record made at the time, no reference should have been made to it. A few days afterward, however, a tumor was observed occupying (as stated by Dr. Trowbridge, who visited the patient again with me) the site of the tumor present at his previous visits. The tumor was large and fluctuating, and after the lapse of a few days more, was opened by Prof. Hamilton, of this city, giving exit to a copious discharge of inodorous pus, which continued to discharge for several months. The abscess beneath the integument communicated freely with the pleural cavity. This case will be referred to again under another division of the subject.

From the history just given, it is suspected that in this case an abscess may have first formed exterior to the chest, which ulcerated in an inward direction, its contents being discharged between the pleural surfaces, giving rise to pleuritis and empyema.

*Quantity of effusion.* Excluding the cases which came under observation after the fluid effused within the pleural cavity had been nearly or quite removed by absorption, together with the cases in which the pleuritis was circumscribed, and the remaining number is *thirty-eight.* Of these thirty-eight cases, the quantity of fluid contained within the chest was relatively *moderate* in *four.* By the term moderate, I would be understood to mean that the chest was less than half filled with the fluid, as determined by physical exploration. The quantity was *considerable* in *eight.* That is to say, the chest was about half filled or somewhat more. The quantity was *large* in *sixteen.* In these cases the chest was full, or nearly so. The quantity *very large* in *ten.* In the latter class of cases the chest was not only filled, but its dimensions enlarged, the heart, in some instances in which the left side was the seat of disease, being pushed beneath, or beyond the right margin of the sternum, and in one instance in which the right side was affected, the liver being depressed below the false ribs.

So far as these observations go, they show that the effusion in general Chronic Pleurisy is never small in amount, but, as a general rule, it is rather large, or very large — a fact which gives to the diagnosis by means of physical signs in all instances simplicity and certainty.

### SYMPTOMATOLOGY.

The record of the symptoms occurring during the career of the disease, in many of the histories, as already stated, is incomplete. In a large proportion, even of the cases which came under observation while the disease was progressing, the disease had already existed for a greater or less length of time. Moreover, the patients did not generally remain constantly under my observation from the date of the first record. In several instances notes at a single examination only were obtained, and sometimes information of the farther progress, and the issue, is wanting, the cases being lost sight of. The number of complete histories is too few to furnish data for determining with precision the numerical ratio in which different symptoms are present or absent in this disease. Nevertheless, an analysis of the facts pertaining to symptomatology which the histories do contain, may be of some practical interest and value.

*Pain.* Most of the histories contain information respecting the amount of pain experienced at the commencement of the disease, and prior to the time that the cases came under my observation. If we exclude the cases in which acute inflammation preceded the chronic form of the disease, and those in which the disease supervened in consequence of perforation, it does not appear that in a single instance pain was severe. In the great majority of cases it was not sufficient in amount to render it prominent as a symptom; in several instances it was altogether wanting. When present it was generally confined to the access, or continued for a short time only after the attack; usually disappearing in the course of a few days. Its character was lancinating, described by patients as stitch-like, and in each instance in which its situation is specified, it was referred to the inferior part of the chest. With respect to the situation, it thus differs from the usual seat of the pains which accompany the ordinary subacute attacks of pleurisy occurring in the progress of tuberculosis. The latter are referred by patients to the upper part of the chest, very frequently beneath the scapula. Severity of pain, approximating to that degree which generally attends the early stage of acute pleurisy, evidently is rarely a symptom of the chronic variety, when the disease is subacute from the beginning; and its presence in ever so slight a degree, is by no means uniform. Without careful inquiry it would often escape the notice of the practitioner in obtaining the previous history of patients affected with Chronic Pleurisy, having been so slight as scarcely to attract attention at the time, or its occurrence been forgotten, and it is therefore mentioned by patients not of their own accord, but only after they have been requested to endeavor to recollect with regard to the point. This is stated in several of the histories in the present collection. Whenever cases come under observation after the disease has existed for several weeks, or months, the absence of pain is the rule. Occasionally a dull pain, or sense of uneasiness in the affected side is complained of; but, so far as my observations go, even these instances are exceptional. The practical bearing of these facts is too obvious to require remark.

*Cough and expectoration.* An examination of the facts noted with respect to these symptoms, leads to the following general conclusions: They are sometimes entirely absent, and are not, therefore, essential to the diagnosis. When present, they are generally not prominent symptoms. This, in fact, so far as these observations go, expresses the rule, the exceptions being very few. Cough and expectoration did not precede the development of the pleurisy except in the few cases in which it was inferred from the history that the patient, prior to the latter disease, was laboring under tuberculosis. The pre-existence of these symptoms, for a considerable length of time, should render the supposition of antecedent tuberculosis highly probable. Pleurisy occurring in patients not tuberculous, is oftener accompanied by cough without, than with an expectoration, or the latter is insignificant in amount. The cough is usually dry, and short or hacking. The expectoration, when present, usually consists of mucus, more or less modified. These facts show that Chronic Pleurisy is often uncomplicated with bronchitis, and if the latter affection coexists, it is of a mild grade.

A copious purulent expectoration sometimes occurs suddenly during the progress of pleurisy, and continues for a greater or less period. Under these circumstances ulceration of the pleura commencing on the free surface of the membrane, and perforation of the lung, opening a communication between the pleural sac and the bronchial tubes may be suspected. This happened in two cases in the present collection, in both instances the pleurisy being circumscribed; and it was supposed to occur in another case after the patient had passed from my observation. The two cases just referred to I shall give in full under another head.

In the cases in which tuberculosis appeared to be developed during the progress of, or subsequent to the pleurisy, cough and expectoration became more or less prominent. Prominence of these symptoms, when before they had been either absent or slight, provided they are not due to perforation, should give rise to strong suspicion of tubercle. This practical consideration is the more important, inasmuch as the physical signs of tubercle are rendered less available by the existence of pleurisy with effusion, and also by the permanent effects of the latter upon the chest.

*Respiration.* Excluding the cases of perforation, the frequency of the respirations was almost uniformly somewhat increased. To this rule, however, there are exceptions. In one of the cases, while the quantity of effusion was very large, removing the heart to the right side of the sternum, the respirations were but sixteen per minute. The increased frequency was not usually great, varying from twenty-five to thirty-five per minute. In one case the respirations were forty per minute. I should remark that the enumeration per minute is noted in but a small proportion of the cases, descriptive expressions being used in the remainder.

The respirations were uniformly increased in frequency by exercise. This was more prominent, as a symptom, than the amount of acceleration while the patients were tranquil. In several instances the chief source of complaint by the patients was, that they were short-winded on walking, or any kind of physical exertion. They would sometimes declare that they were quite well, and able to labor except for this difficulty. The want of breath was also apparent in conversation. The patients were obliged to stop frequently in the midst of sentences to inspire.

Dyspnœa, or a painful sense of breathlessness, does not appear to have been present in any of the cases, while the patients were at rest. In cases in which the chest was filled with liquid effusion, the respirations rendered short and frequent by slight exercise, and imperfect oxygenation of the blood denoted by lividity of the prolabia, and other parts, there was not a marked degree of suffering from dyspnœa. In one of the cases only, is it noted that this symptom was in any measure prominent. This was a case of empyema in which the purulent contents of the chest having made their way through the thoracic walls were evacuated externally. In this case, for a time, the dyspnœa was considerable, causing the patient to preserve the semi-erect position. It was, however, relieved before the discharge of the contents of the chest took place. It is possible that dyspnœa may have occurred in other of the cases while they were not under my observation; but it may be safely stated that they show the absence, or non-prominence of this symptom to be the rule in Chronic Pleurisy.

*Circulation.* The pulse was found to be more or less accelerated, in the great majority of the cases, during the progress of the disease. The degree of acceleration differed considerally in different cases, ranging usually from 80 to 120, but in one case reaching 140 and in another 160 per minute. In a few instances, at the time the symptoms were noted, the pulse preserved its normal frequency, and in one instance was below the average standard, being sixty-four per minute. As respects other characters, in all the histories containing information on this point, the pulse was small, compressible, and sometimes quite feeble. It would be highly interesting to study the pulse, as well as other symptoms, in the different classes of the cases of Chronic Pleurisy separately: for examples, in the cases characterized by the coexistence of tubercle, and in which this complication was absent; in cases in which the liquid effusion became purulent, constituting empyema, etc. The present data, however, are inadequate for the prosecution of these inquiries to an extent sufficient to render them of much value.

Lividity of the prolabia is noted in three cases, all of which proved fatal, and in two the disease was complicated with pericarditis.

In the history of one case in which marked capillary congestion of the surface of both extremities was observed, the left upper extremity presented this appearance to a greater extent than the right, and the temperature was sensibly less. In this case the left side was the side affected, and the amount of effusion was large.

*Skin.* Sweating is an event occurring pretty often in this disease. The sweating is frequently profuse, and is most apt to occur at night. The sweating is by no means uniformly preceded by a febrile paroxysm, or exacerbation. I think it probable that in but few, if any of the instances, was well marked hectic present, but on this point the histories are defective. To be precise with respect to sweating, it is noted, as a prominent symptom, in eleven of the cases. Of the remaining cases it is noted to have not been present in a few, but in most of the cases nothing is stated relative to it.

Aside from sweating, the skin is stated to have been cool, or cool and moist in several of the cases, but in a large proportion the condition of the surface is not noted.

*Chills.* In a small proportion of cases a chill, or chills, or chilly sensations are noted to have occurred. Here, as with respect to other symptoms, my data do not authorize the deduction of an exact numerical ratio. Chills may have been present in some cases after the periods in the progress of the disease to which my records extend, or they may have escaped attention in recording the previous history; in the latter instance they would not have constituted a very prominent symptom. In the cases in which chills are noted to have occurred, they do not appear to be uniformly associated with other events investing them with any special significance. They occurred in cases in which the progress and issue of the disease showed that the liquid effused within the chest did not become purulent; and also in cases in which there were no evidences of coexisting tuberculosis. In one case the recurrence of chills for several successive days preceded a sudden moderate purulent expectoration which continued but for a short time. This patient recovered, and has, for several years, been in good health. In two cases, the chills were so prominent in the early part of the disease that the patient was supposed to be laboring under intermittent fever.

*Digestive System.* Anorexia, or impaired appetite, enter into the history of many of the cases, but it is remarkable how small was the disturbance of the digestive system in several instances. The appetite was tolerable, and sometimes excellent, and the digestion good, during the whole progress of the disease. In several of the histories it is noted that the tongue was perfectly clean. A few cases were characterized by the occurrence of diarrhœa' not colliquative, but apparently exerting a salutary effect, being followed by diminution in the amount of the liquid effusion within the chest.

*Aspect and nutrition.* The countenance presented, in some cases, marked pallor, but in other cases the aspect was not notably morbid, and, in some instances, while the quantity of liquid effusion within the chest was considerable, the general appearance denoted good health.

Progressive emaciation characterized the cases ending fatally, but of those in which recovery took place, the loss of weight was not always great, and in several instances considerable embonpoint was preserved.

*Strength.* The muscular strength was retained in a remarkable degree by some of the cases terminating fatally, the patients being able to sit up until within a short time before death. In one case, particularly, this preservation of strength was exhibited in a striking degree. The patient was considerably emaciated, there were anorexia, short and labored respiration, incessant hacking cough, coldness of extremities and œdema, lividity at the roots of the nails, frequent and intermitting pulse — these symptoms preceding dissolution — but he was still able to sit up, and insisted on being dressed. In several cases in which the quantity of liquid effusion was large, filling the chest, the patients were not confined to the bed at any time during the progress of the disease; but under these circumstances, in some instances, they were able to be out of doors, taking exercise and even performing a considerable amount of labor. For example, in the case of a patient who applied to me six weeks after the date of attack, having had no medical advice up to that time, the right chest was completely filled with fluid. He had, however, not kept the bed even for a single day, but had continued steadily in his occupation, embracing wood-sawing, taking care of a horse, and the varied duties of a house servant. He rapidly recovered after ceasing labor, being at no time prevented from going out of doors, and walking always a mile to my residence for medical advice.

As another illustration, a lad, aged ten years, had labored under the disease for two months before the diagnosis was made. During that time he had never been kept at home, but had daily been accustomed to play out of doors. He was allowed to continue his out-door sports, and he progressively improved and recovered, complaining only of being put out of breath on active exercise. At the time of the examination, two months after the date of the attack, the left chest was completely filled with liquid effusion.

*Urine.* In a few cases in which the condition of the urine was ascertained, it was uniformly scanty and high colored. The information does not extend beyond the qualities just mentioned.

Retention of urine, requiring the catheter for several days, was a symptom in one case.

*Œdema.* Œdema of the lower extremities is noted in the histories of three cases, and in one of these cases the face was also œdematous. Two of the cases terminated fatally.

*Hœmoptysis.* This symptom was present in two cases. In one of the cases, it preceded, together with slight cough, the development of the pleurisy. The history renders it altogether probable that tubercle coexisted in this case. In the other case the hœmoptysis occurred twice in the early part of the disease. The patient, however, progressed favorably, and three years afterward, was in good health, being free from any pulmonary symptoms, except want of breath for active exercise. A few months afterward he had a third attack of hæmorrhage, which was quite copious. This was not followed by any new pulmonary symptoms, and at the present time, about four months after the last attack of hœmoptysis, he is actively engaged in business, is free from cough, and considers himself quite well. It is, however, by no means certain that the lungs are free from tubercle in the latter case.

### PHYSICAL SIGNS.

The physical signs, in most of the cases, were determined only so far as was required to establish the diagnosi. They were not recorded, more than the symptoms, with a view to numerical analyses. I shall consequently give, in this division of the subject, as in that devoted to the symptomatology, the practical results of an examination of the facts contained in the histories, without aiming to ascertain the relations which the signs individually bear to each other, and the disease, as respects the ratio of their occurrence. In the following account I will exclude reference to the cases of perforation, of circumscribed pleurisy, and those in which the disease had existed at a period more or less removed from the time the cases came under my observation. These three groups of cases will presently be considered under distinct heads, and attention will therefore now be directed only to the cases of ordinary chronic, general pleurisy, during the progress of the disease.

*Percussion.* The signs developed by percussion, were quite uniform, viz., flatness extending from the bottom of the chest upward, either entirely, or to a greater or less extent over the side affected; resonance clear, apparently exaggerated, over the healthy side, and in cases in which the flatness was not universal over the affected side, relative dullness of resonance above the upper boundary of the flatness, compared with the corresponding portions of the healthy side.

The few deviations from, or additions to the above rule of the percussion signs, which are noted, are as follows:

In one case the resonance was tympanitic over the lower third of the left side, which was the side affected, a tympanitic resonance also being present over the epigastrium. This was undoubtedly due to flatulent distension of the stomach.

The flatness in two cases was observed to extend from the side affected, beyond the sternum, over a small portion of the healthy side. In both cases the quantity of liquid effusion was very large, distending the chest in every direction, and pushing laterally the mediastinum so as to encroach on the opposite half of the chest.

In two cases the resonance on the affected side above the line of flatness, was exaggerated, the clearness being relatively greater, in a marked degree, than on the healthy side. In one of these cases it is noted that the clearness of resonance was apparent over the upper third anteriorly and posteriorly. The chest on that side was enlarged, intercostal depressions lost, motions in respiration limited, and there was absence of all respiratory sound over the inferior and middle thirds. The respiration over the upper third was feeble, the vesicular quality absent, and the expiration prolonged. The greater relative clearness of resonance, at the summit of the chest on the left side, continued, and after the lapse of a month, the marked contraction of the affected side having taken place, the same disparity between the two sides was noted.

In the other case the infra-clavicular region of the affected side was bulging, and in this region the resonance was clearer than in the corresponding region on the healthy side. The respiration was relatively feeble; its qualities not being described. Flatness on the affected side existed below the second rib. The side (which was the left) was visibly enlarged, measuring one-fourth of an inch more than the right. In one case there was flatness over the left side *except posteriorly at the inferior portion of the chest below the angle of the scapula*, tubular respiration was heard over the situation just mentioned. The left side was comparatively immovable. The heart was carried upward and to the right, the point of impulse being perceptible between the nipple and left margin of the sternum.

Tenderness apparent on percussion, is noted in several of the histories.

It does not appear that a change in the level of the liquid contents of the chest, in different positions of the patient, was observed in any instance, but I am not certain that pains were taken, generally, to examine for this sign, which is frequently found in cases of acute pleurisy and of hydro-thorax. The great amount of effusion in a large proportion of cases of Chronic Pleurisy renders it often unavailable.

*Auscultation.* It will suffice with respect to this, as well as the other classes of physical signs, to notice any peculiarities differing from the ordinary rule in this disease. The rule of auscultatory signs in Chronic Pleurisy, is as follows: Absence of all respiratory sound on the affected side from the bottom of the chest upward, as far as the flatness on percussion extends; respiration above this line on the affected side more or less developed, and tubular and bronchial in character, with bronchophony in some cases; occasionally œgophony; on the healthy side the respiratory sound normal in character, and increased in intensity, constituting the respiration known as exaggerated, supplementary or puerile.

On examination of the facts contained in the histories, after writing the foregoing paragraph, I find but a single case in which any anomalous deviation from the usual signs is noted. In this case a faint and apparently distant bronchial respiration was heard generally over the chest on the affected side. There was flatness over this side except at the summit. The side was visibly enlarged, but no marked disparity in mobility. This was the case, already referred to, in which there was bulging of the infra clavicular region on the affected side, and a greater amount of resonance in that situation, than in the same region on the healthy side.

A friction sound was heard in three cases. In one of these cases it existed early in the disease before much liquid effusion had taken place. In another it was noticed by the patient himself, after the disease had existed for some time, and he was able to be about, having been for some time confined within doors. In the remaining case it existed during the progress of the disease, while the quantity of effusion was considerable. These were all the cases in which this sign was discovered, but it was not always carefully sought for, at different stages of the disease, in the cases individually.

Œgophony was not often sought for. It was not distinctly noticed in any instance, but in one case there was an approach to it, the voice having a œgophanous modification.

Bronchophony was observed in several of the cases at the summit of the chest on the affected side.

Adventitious sounds of respiration, or râles, are not noted to have been present in but two cases. In one of these cases sibilant and sonorous râles were heard at the summit of the chest on both sides; and in the other case crackling in the same situation. In both cases the history of the case showed the coexistence of tubercle.

*Inspection.* Visible enlargement of the chest on the affected side was a well marked sign in several cases. Permanent expansion of the affected side, rendering it comparatively immovable in respiration, was usually observed in the cases in which the amount of liquid effusion was large. The intercostal spaces, except in some instances in which the quantity of adipose tissue prevented depressions from being apparent, were elevated to the level of the ribs, and in some cases projected beyond this level. The contrast between the two sides in this respect, was frequently striking.

After the liquid effusion was absorbed, or considerably lessened, contraction of the chest was apparent in all the cases which remained under observation. The diminished semi-circumference on the affected side was frequently obvious; but the change was more marked in the depression of the shoulder, and generally, but not invariably, in the diminution of the interscapular space.

Œdema of the affected side is noted to have existed in some cases, and in other cases its absence is stated, but a large proportion of the histories contain nothing relative to this point. The same is true of enlargement of the subcutaneous views on the affected side.

*Mensuration.* Measurement showed, in several cases, the affected side to be distended while the amount of liquid contents was large; and afterward contraction to less than the normal size. The greatest increase in any of the cases in which measurement was practiced, was two inches. The greatest degree of contraction noted is three-fourths of an inch, but mensuration was not resorted to, after absorption was completed, in some instances in which the contraction was probably greater than this.

*Palpation.* The absence on the affected side of a vocal fremitus, appreciable on the healthy side, is noted in several of the histories. In two cases a sense of fluctuation was perceived in the intercostal spaces.

*Displacement of the Heart.* In the cases in which the disease was seated in the left side, and the quantity of liquid effusion large, the heart was displaced, to a greater or less extent, from its normal situation. This was shown, in some instances, by the impulse not being felt at an[illegible]int, the organ being too far removed from the walls of the chest, to be brought into collision by the systolic contraction. Under these circumstances the cardiac sounds were heard over a considerable space, but usually rather feeble and distant. This rule is not without exceptions. In one instance the sounds of the heart were heard with considerable intensity over the chest, and a shock communicated to the walls of the affected side, so that the attending physician, who was but little acquainted with physical exploration, supposed the case to be one of disease of the heart.

The displacement, in other instances, was rendered more obvious by the impulse being appreciable at a greater or less distance from the point where it is felt in health. In five cases the impulse was felt or seen beyond the right margin of the sternum. The quantity of fluid, in these cases, was sufficient to push the heart and mediastinum laterally, causing them to occupy a portion of the space belonging to the right side of the chest. The heart was removed in an upward as well a lateral direction in these cases, the impulse being apparent in the right mammary region in one case, and between the third and fourth ribs in another case. In the instances in which the displacement was not so great, the impulse was perceived at different points in a line running directly in an upward and lateral direction to the right. As the liquid contents of the chest diminished by absorption, the heart receded along the same line in a direction toward its normal position.

In the cases in which the right side was the seat of the disease, displacement of the heart is noted in but one instance. Pains were, perhaps, not generally taken to ascertain whether the relations of the organ were disturbed, as I find nothing relative to this point in the histories, save in the single instance referred to. In that instance the patient came under observation four months after the date of attack. The right chest then presented marked contraction, and diminished mobility. There was flatness on the right side below the nipple, and absence of respiratory sound. The impulse of the heart was found on a level with the left nipple and between the sternum. This displacement was probably due to the absorption of the fluid effused into the right chest, a result of Chronic Pleurisy on that side which was first noticed by Dr. Stokes, of Dublin.*

---

* This Report will be continued.

